# Pharmacy students’ provision of health promotion counseling services during a community pharmacy clerkship: a cross sectional study, Northwest Ethiopia

**DOI:** 10.1186/s12909-018-1216-0

**Published:** 2018-05-04

**Authors:** Dessalegn Asmelashe Gelayee, Gashaw Binega Mekonnen

**Affiliations:** 1 Department of Pharmacology, College of Medicine and Health Sciences, University of Gondar, P.O.Box 196, Gondar, Ethiopia; 2 Department of Clinical Pharmacy, College of Medicine and Health Sciences, University of Gondar, P.O.Box 196, Gondar, Ethiopia

**Keywords:** Clerkship, Counseling, Ethiopia, Health promotion, Pharmacy, Students

## Abstract

**Background:**

Globally, undergraduate pharmacy education comprises practice programs aimed to address different competencies. This study was intended to investigate pharmacy students’ provision of health promotion (HP) counseling services during a community pharmacy clerkship in Northwest Ethiopia.

**Methods:**

A prospective cross-sectional study was conducted on fifty one fifth-year pharmacy students immediately after completion of a 2-week community pharmacy clerkship. Data were collected through a self-administered questionnaire. Relationship between variables was examined using Pearson’s Chi-square test of independence, Mann–Whitney U test, and Spearman’s rank correlation coefficient.

**Results:**

The mean number of HP counseling service types delivered during the clerkship was 6.3 ± 2.8 out of 12. It is positively correlated with the number of HP counseling service types delivered in students’ previous training (rho =0.437, *p* = 0.001). Nearly half (*n* = 25, 49%) of the students were actively-involved (i.e delivered ≥ 7 types of HP counseling service types) in the service and those who were well involved in previous training are more likely to do the same during the clerkship (*X*^*2*^ = 4.581, *p* = 0.032). The main barriers perceived to hinder health promotion service were clients’ lack of time and interest as well as absence of a guideline for health promotion service.

**Conclusion:**

Community pharmacy clerkship is a good opportunity for pharmacy students to develop health promotion counseling skill. Clerkship performance can best be improved through successful exposures to similar activities in previous courses and students shall be encouraged to carry out self-assessments of their health promotion counseling practice against standards set for the clerkship.

**Electronic supplementary material:**

The online version of this article (10.1186/s12909-018-1216-0) contains supplementary material, which is available to authorized users.

## Background

Pharmacists’ role in patient care continues to grow along with the profession. Thus undergraduate pharmacy education has been changed in many ways. Practice programs account for a significant part of these changes [1]. Pharmacists are required more than ever to contribute in the area of health promotion (HP), and it is one of the six components that contribute to the health improvement of individuals accessing pharmacy services as stated in the Joint FIP/WHO guideline on good pharmacy practice [2]. The importance of the role of pharmacists in patient counseling is recognized [3] and because of increased accessibility, they are in a key position to provide HP services [4]. Several studies have shown the benefits of pharmacists’ involvement in a wide range of important public-health issues including smoking cessation, diabetes, hypertension and contraception [5]. However most pharmacy schools in Africa do not ingrain public health concepts including health promotion in their curricula. Thus review of the the curricula and teaching methodology seems vital [6]. In Ethiopia, the minimum entry level for pharmacy practice is diploma (for pharmacy technicians) and they are qualified to run drug stores. Whereas, community pharmacies need to be run by a pharmacist with at least a Bachelor degree in Pharmacy. This is a 5-year program with final year allocated for a clerkship programme. The nationally harmonized curriculum incorporates a Community Pharmacy clerkship course of 5 ECTS points during the fifth year [7]. The nationally harmonized curriculum stated that one of the objectives to be met in the clerkship is provision of public health and wellness services tailored to the needs of patients.

On the other hand, non-commuicable diseases (NCDs) have become a major public health concern in the nation accounting for 30% of deaths in 2014 alone. Eighty percent of this is accounted for by cardiovascular diseases, cancers, diabetes and chronic respiratory diseases [8]. The shared common risk factors are tobacco, insufficient physical activity, unhealthy diet and excessive alcohol use which are preventable [8]. Pharmacists can join the campaign against such risks through health promotion services. Workye et al. [9] and Ayalew et al. [10] have shown that the majority of clients in Gondar town, Northwest Ethiopia expect health promotion services from pharmacies such as life style modifications required for the underlying medical condition. The importance of teaching student pharmacists how to implement and deliver wellness and prevention services has been recognized [11]. Early exposure to health promotion and disease prevention concepts may help strengthen students’ desire and expectations to fulfill these expanded pharmacy roles [12].

The future of the pharmacy profession is in the hands of todays students. Thus student pharmacists must be taught how to provide the health promotion service. This is especially so for community pharmacists who are very accessible to the community and hence are an in an ideal setting to provide the sevice. Community Pharmacy Clerkships in particular, need to boldly address the issue of developing student health promotion counseling skill in the settings of community pharmacies. Then evaluation and assessment of the clerkship outcomes would have much greater implications for health educators and authorities.

This study therefore aimed to investigate health promotion counseling practice, its quality and the barriers to provision of the service, as reported by pharmacy students during a 2 week community pharmacy clerkship. The study also aimed to determine is there was any ‘bottleneck’ to developing students as health promotors, in pharmacist education.

## Methods

### Study design and setting

This prospective cross-sectional study was conducted during December 2016 among pharmacy students of University of Gondar. It is a public university located in Gondar, Northwest Ethiopia and offers Bachelor of pharmacy education in both regular and continuing education program. All clerkship students of 2016/17 were assigned to community pharmacies (6 students per facility on average) which are run by a B.pharm graduate and all of which were at the same level. Drug stores which are run by pharmacy technicians were not used to train the students in the clerkship.

### Study population

All 51 undergraduate fifth year pharmacy students enrolled in the regular program of University of Gondar in 2016/17 were involved in the study. This population was purposely selected because they were final year students who have completed all the pre-requisite courses to practice in a Community Pharmacy Clerkship, and were thought to have assimilated the knowledge and skill of counseling. They were the best student population for an evaluation of the counseling practice of pharmacy undergraduate students.

### Data collection procedure

The students were contacted immediately after completion of the 2 weeks clerkship and data were collected through a self-administered questionnaire prepared based on previous studies [13, 14] with some modifications (see Additional file 1).

It consisted of 3 main parts: Previous involvement in HP counseling services; Involvement in HP counseling services during the clerkship; Barriers that limit the practice.

The questionnaire was validated for its content by three senior pharmacologists and pretested on 10 clerkship students enrolled in the continuing education of the same university. Necessary modifications were made before distributing the questionnaires to the students. The reliability of the different sub-components of the questionnaire was measured and the Cronbach’s alpha value was 0.892 (involvement in health promotion counseling services during previous training, 12 items), 0.798 (health promotion counseling services delivered during community pharmacy clerkship, 12 items), and 0.766 (barriers for health promotion counseling service provision during the clerkship, 6 items).

### Data analysis

The data were entered into computer for analysis by the statistical package for social sciences (SPSS) version 20.0 for windows (SPSS Inc., Chicago, Illinois). Descriptive statistics, Mann–Whitney U test, Pearson’s Chi square test of independence and Spearman’s rank correlation coefficient were employed in the data analyses. Results are assumed to be significant at *p* < 0.05.

### Ethical considerations

All respondents were asked for their verbal consent as approved by Ethical Review Committee of the School of Pharmacy, University of Gondar. Only the aggregate data was used for research purpose.

## Results

This prospective cross sectional study was conducted among undergraduate fifth year pharmacy students in a public Ethiopian University. It describes health promotion counseling practice and the barriers that students faced in providing that service during a Community Pharmacy Clerkship.

### Socio-demographic characterstics and additional responses

As shown in Table 1, the majority of them were male (*n* = 32, 62.7%) and the mean age was 22.63 ± 1.02 years with minimum of 20 and maximum of 25 years. The overall pharmacy training curriculum of the university was perceived to be adequate to prepare graduates to render health promotion counseling service (*n* = 39, 76.5%). There is no significant difference between male and female students with regard to this opinion. Only 29 (56.9%) students received a course syllabus for the Community Pharmacy Clerkship ahead of the practice.

**Table 1:** Socio-demographic characterstics and additional responses (*N* = 51)

Variables	N (%)
Gender	
Female	19 (37.3%)
Male	32 (62.7%)
Age mean = 22.6 ± 1.0	
Training curriculum is adequate for offering health promotion service	
Yes	39 (76.5%)
No	12 (23.5%)
Received course syllabus for the clerkship	
Yes	29 (56.9%)
No	22 (43.1%)

### Health promotion counseling practices during the clerkship

**Fig. 1:**
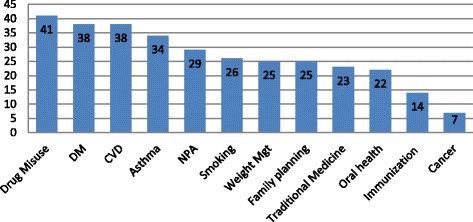
Health promotion counseling practices during community pharmacy clerkship (*n* = 51). DM: Diabetes Mellitus; CVD: Cardiovascular Disorder; NPA: Nutrition and physical activity; Mgt: management

Of the 12 different types of health promotion counseling services assessed, the mean number of service types delivered during the 2 weeks Community Pharmacy Clerkship was 6.3 ± 2.8, min = 0 and max = 12. The mean for females (6.0 ± 2.2) and mean for males (6.5 ± 3.1) were not significantly different (*p* = 0.517) in the Mann-Whitney U test. As shown in Fig. 1, the most commonly delivered services were counseling related to drug misuse, diabetes mellitus and cardiovascular diseases. However, cancer and immunization related services were reported to be the least. Nearly half (*n* = 25, 49%) of the students were actively involved in the service (i.e delivered ≥ 7 types of health promotion counseling services) during the clerkship.

### Health promotion counseling practices during previous study years

**Fig. 2:**
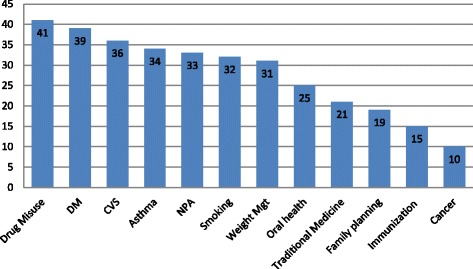
Health promotion counseling practices in previous study years (*n* = 51). DM: Diabetes Mellitus; CVD: Cardiovascular Disorder; NPA: Nutrition and physical activity; Mgt: management

A similar pattern of health promotion service was reported by the students in previous years of study. The mean number of service types given was 6.6 ± 2.7, min = 0, max = 12. The mean of females (6.8 ± 2.8) and mean of males (6.5 ± 2.7) were not significantly different (*p* = 0.679) in the Mann-Whitney U test, as shown in Fig. 2.

There was a significant positive correlation between the number of HP service types practiced in previous years and those delivered in the Community Pharmacy Clerkship (rho =0.437, *p* = 0.001). The proportion of students actively involved in health promotion service during the clerkship was 49% (*n* = 25) while that for previous training was 56.9% (*n* = 29). No significant difference was found between females and males in being actively involved during either the clerkship (*p* = 0.447) or previous training (*p* = 0.484). However, those who were actively involved in HP services previously were more likely to be actively involved during community pharmacy practice (*X*^*2*^ = 4.581, *p* = 0.032).

### Perceived quality of HP counseling practice during the clerkship and satisfaction of the students

Only 22 (43.1%) students perceived that the quality of the health promotion service delivered in the community pharmacy clerkship, was of good quality. The same proportion of students (43.1%) reported that preceptor quality in coaching for health promotion during the clerkship was good, while 5 (9.8%) rated preceptor quality as very good (See Table 2). About 31 (60.8%) students were satisfied/very satisfied with the service they provided. The satisfaction of students with the service provided was positively correlated with the quality of the service (rho = 0.398, *p* = 0.004).

**Table 2:** Perception of quality of health promotion counseling practice and satisfaction with the service provided (*N* = 51)

Variable	Response			
	Poor	Fair	Good	Very good
Quality of health promotion service provided	7 (13.7%)	22(43.1%)	22(43.1%)	0
Preceptors’ effort to introduce students in health promotion services	7 (13.7%)	17(33.3%)	22(43.1%)	5 (9.8%)
	Very unsatisfied	Unsatisfied	Satisfied	Very satisfied
Satisfaction with the health promotion service students provided	3 (5.9%)	17(33.3%)	30(58.8%)	1 (2%)

4 point likert scale (1 = poor/very unsatisfied, 4 = very good/very satisfied)

### Perceived barriers to provide health promotion counseling during the clerkship

Only 28 (54.9%) students reported that they observed health promotion services being delivered in the community pharmacy in which they were assigned to practice. As shown in Table 3, the main barriers (agree/strongly agree) hindering health promotion service were lack of time (*n* = 32), lack of clients’ interest (n = 32) and absence of a guideline for health promotion service (*n* = 27). Based on the Mann-Whitney U test, no group difference was observed based on gender of the respondents.

**Table 3:** Barriers that limit students’ involvement in health promotion cousseling practice (*N* = 51)

Barrier	Response			
	SD	D	A	SA
Lack of time by clients	5 (9.8%)	14 (27.5%)	27 (52.9%)	5 (9.8%)
Lack of interest by clients	5 (9.8%)	14 (27.5%)	25 (49%)	7 (13.7%)
Absence of standard guideline for the services	10 (19.6%)	14 (27.5%)	18 (35.3%)	9 (17.6%)
Lack of training/knowledge	9 (17.7%)	17 (33.3%)	20 (39.2%)	5 (9.8%)
Lack of confidence	10 (19.6%)	20 (39.2%)	19 (37.3%)	2 (3.9%)
I have no interest	22 (43.1%)	18 (35.3%)	8 (15.7%)	3 (5.9%)

*SD* strongly disagree, *D* disagree, *A* agree, *SA* strongly agree

## Discussion

The findings of the present study indicated that Community Pharmacy Clerkship is an important means for developing the skills of undergraduate pharmacy students on health promotion counseling practices. Yet, there is a perception that it lacks diversity and quality. The main barriers for the practice were lack of time and interest among clients and absence of a guideline for health promotion counseling practice. The finding highlights for health educators and authorities the importance of revising the curriculum and its delivery.

### Health promotion counseling practice

A limited number of studies have identified different health promotion counseling topics for community pharmacists in Africa: Asthma; Diabetes; Cardiovascular Disease; Drug misuse; Nutrition and Physical Activity; Smoking Cessation; Oral health; Immunization; Traditional Medicine; Weight Management; Family Planning; and Cancer [14]. Other studies have reported public health roles of community pharmacists in Ethiopia such as counseling on alcohol consumption and on healthy eating [15] as well as counseling on traditional medicines [16] and oral health [17]. Community pharmacists in Africa seem to have a positive attitude towards public health roles such as health promotion counseling [14, 18].

The contemporary curriculum for Bachelor of Pharmacy in the University of Gonadr was perceived to be sufficient enough by the majority of students to prepare them to deliver health promotion services. However this is in contrast with the report that concepts of public health are hardly entertained in the curricula of most pharmacy schools in Africa [6]. It is encouraging that nearly half of the HP topics included in the survey were delivered during the clerkship with no statistical difference between males and females (*p* = 0.517). The fact that the most used topics were drug misuse, diabetes mellitus as well as cardiovascular diseases, could be due to the higher proportion of clients visiting the pharmacies requesting medications useful in cardiovascular disorders and diabetes mellitus. Both of these diseases are among the major NCDs linked to high mortality in Ethiopia [8]. The finding highlights that the diversity of health promotion topics covered in the clerkship is limited. It is in contrast to a previous study where lots of health promotion topics were covered [14]. Specially, cancer related health promotion service was the least performed and students need to be well trained to improve this service since cancer accounts for a significant share of mortality related to NCD [8].

Overall, the majority of students were not actively involved in HP service during the clerkship. In the era of more patient focused pharmacy profession [19], students need to be trained very well to enhance their involvement in HP service. Previous experience and current practice of HP counseling were positively correlated (*p* = 0.001) highlighting the importance of diversifying and strengthening practice of students in the study years prior to the clerkship. Quality education should be built from the bottom during pre-service training. However, this contrasts the findings of Offu et al. [18] where community pharmacists’ public health practice in Nigeria was independent of previous experience.

### Perceived quality and barriers of health promotion counseling practice during the clerkship

As the majority of students rated the quality of HP service they delivered and the preceptors’ quality in training health promotion poorly, much emphasis shall be given to build capacity of preceptors and properly monitor students’ performance in clerkships to meet the desired objectives. If pharmacy education is to be improved, preceptors are also responsible for improving their coaching skills [20–22].

The satisfaction of students with the service they provided was positively correlated with the quality of the service (*p* = 0.004). Professional attitudes of students may be influenced by such poor practice experiences and much emphasis shall be given to promote quality practice. Observations of practicing pharmacsists who were less involved in HP counseling service might have its own negative influence on students’ practice. In the present study, nearly half of the students did not observe HP services in the practice site. Thus, it seems important to improve the practice of pharmacists if they are to successfully support the clerkship and participate as potential preceptors.

Several factors have been identified as barriers to practice health promotion in community pharmacy settings such as lack of time, lack of training, and absence of standard practice guideline [5, 13–15, 23]. The barriers identified in the present study are consistent with these studies. In this regard we suggest further studies to assess preceptors’ competency and commitment in monitoring such clerkships. The reasons why clients are not interested with health promotion services requires further assesment. It would sometimes be important only to sensitize clients on health promotion topics and ask them to come back some other time.

This is a baseline study and a pioneer in the nation. Although it may not apply to all pharmacy students in Ethiopia, it gives an insight into health promotion counseling practice of pharmacy students in a developing country. The present study is not without limitations. The fact that the community pharmacy clerkship was only of 2 weeks duration might affected the number of health promotion topics covered by the students and thus undermined the finding in the present study. Since it is a retrospective study and only assesses sudents’ perception, qualitative and observational studies need to support its findings. There could be some response bias as the questionnaire was self-administered and the small sample size may also limit generalizability of our findings.

## Conclusion

The findings of the present study showed that Community Pharmacy Clerkship is a good opportunity for pharmacy students to develop health promotion counseling skill. We recommend considering curricula revision to make sure that health promotion services are boldly addressed in the clerkship as well as in other courses. In addition, it would be important to engaging students in self-assessments of their health promotion counseling practice during the clerkship according to a well established standard of practice developed locally. Improving the competency and commitment of instructors and community pharmacists likely will improve the outcomes of a community pharmacy clerkship.

## Additional file

Additional file 1: Health Promotion Counseling Survey Questionnaire. It consists of four main parts on sociodemographic characterstics, health promotion counseling services in previous clerkships as well as the community pharmacy clerkship and barriers that limit the practice. (DOCX 19 kb)

## Abbreviations

HP: Health Promotion
NCD: Non-communicable Diseases
