# Supplementary material for: Pharmacy students’ provision of health promotion counseling services during a community pharmacy clerkship: a cross sectional study, Northwest Ethiopia
Source: BMC Med Educ. 2018 May 4;18:95. doi: 10.1186/s12909-018-1216-0 (PMC5935927; doi:10.1186/s12909-018-1216-0)
Supplement: Supplementary file 1 — Health Promotion Counseling Survey Questionnaire. It consists of four main parts on sociodemographic characterstics, health promotion counseling services in previous clerkships as well as the community pharmacy clerkship and barriers that limit the practice. (DOCX 19 kb) [file 12909_2018_1216_MOESM1_ESM.docx]

**Pharmacy students’ provision of health promotion counseling services during a community pharmacy clerkship**

**Socio-demography**

1. Sex: female male
2. Age (yr):________
3. Do you think your training curriculum is adequate for offering health promotion service? yes no
4. Did you receive course syllabus for community pharmacy clerkship? yes no
5. **Involvement in health promotion counseling service during previous clerkships (other than community pharmacy clerkship)**

| **Activities** | **Level of involvement** | | | |
| --- | --- | --- | --- | --- |
|  | **Very uninvolved** | **Uninvolved** | **Involved** | **Very involved** |
| 1. Asthma counseling |  |  |  |  |
| 1. Diabetes counseling |  |  |  |  |
| 1. Cardiovascular counseling |  |  |  |  |
| 1. Drug misuse |  |  |  |  |
| 1. Nutrition and physical activity |  |  |  |  |
| 1. Smoking cessation counseling |  |  |  |  |
| 1. Oral health |  |  |  |  |
| 1. Immunization |  |  |  |  |
| 1. Traditional medicine counseling |  |  |  |  |
| 1. Weight management counseling |  |  |  |  |
| 1. Family planning |  |  |  |  |
| 1. Cancer counseling |  |  |  |  |

1. **Types of health promotion counseling services provided during community pharmacy clerkship**

| **Activities** | **Response** | |
| --- | --- | --- |
|  | **yes** | **no** |
| 1. Asthma counseling |  |  |
| 1. Diabetes counseling |  |  |
| 1. Cardiovascular counseling |  |  |
| 1. Drug misuse |  |  |
| 1. Nutrition and physical activity |  |  |
| 1. Smoking cessation counseling |  |  |
| 1. Oral health |  |  |
| 1. Immunization |  |  |
| 1. Traditional medicine counseling |  |  |
| 1. Weight management counseling |  |  |
| 1. Family planning |  |  |
| 1. Cancer counseling |  |  |

1. How do you evaluate the quality of health promotion service you provided? poor fair good very good
2. How do you rate your satisfaction with the health promotion service you provided?

very unsatisfied unsatisfied satisfied very satisfied

1. How do you evaluate preceptors’ effort to introduce/engage you in health promotion services?

poor fair good very good

1. Did you observe health promotion services being delivered in the community pharmacy you were assigned to practice?

yes no

1. **Barriers that limit your involvement in health promotion services**

| **Barrier** | **Response** | | | |
| --- | --- | --- | --- | --- |
|  | **SD** | **D** | **A** | **SA** |
| 1. Lack of time by clients |  |  |  |  |
| 1. Lack of interest by clients |  |  |  |  |
| 1. I have no interest |  |  |  |  |
| 1. Lack of training/knowledge |  |  |  |  |
| 1. Lack of confidence |  |  |  |  |
| 1. Absence of standard guideline for the services |  |  |  |  |
| **Others (specify)** | | | | |
| **SD=strongly disagree;D=disagree;A=agree; SA=strongly agree** | | | | |
